# Supplementary material for: Reprogramming CAR T-Cells with designed bioPROTACs
Source: bioRxiv. 2026 Feb 22:2026.02.21.706835. Preprint. [Version 1] doi: 10.64898/2026.02.21.706835 (PMC12934639; doi:10.64898/2026.02.21.706835)
Supplement: Supplement 4 [file media-4.pdf]

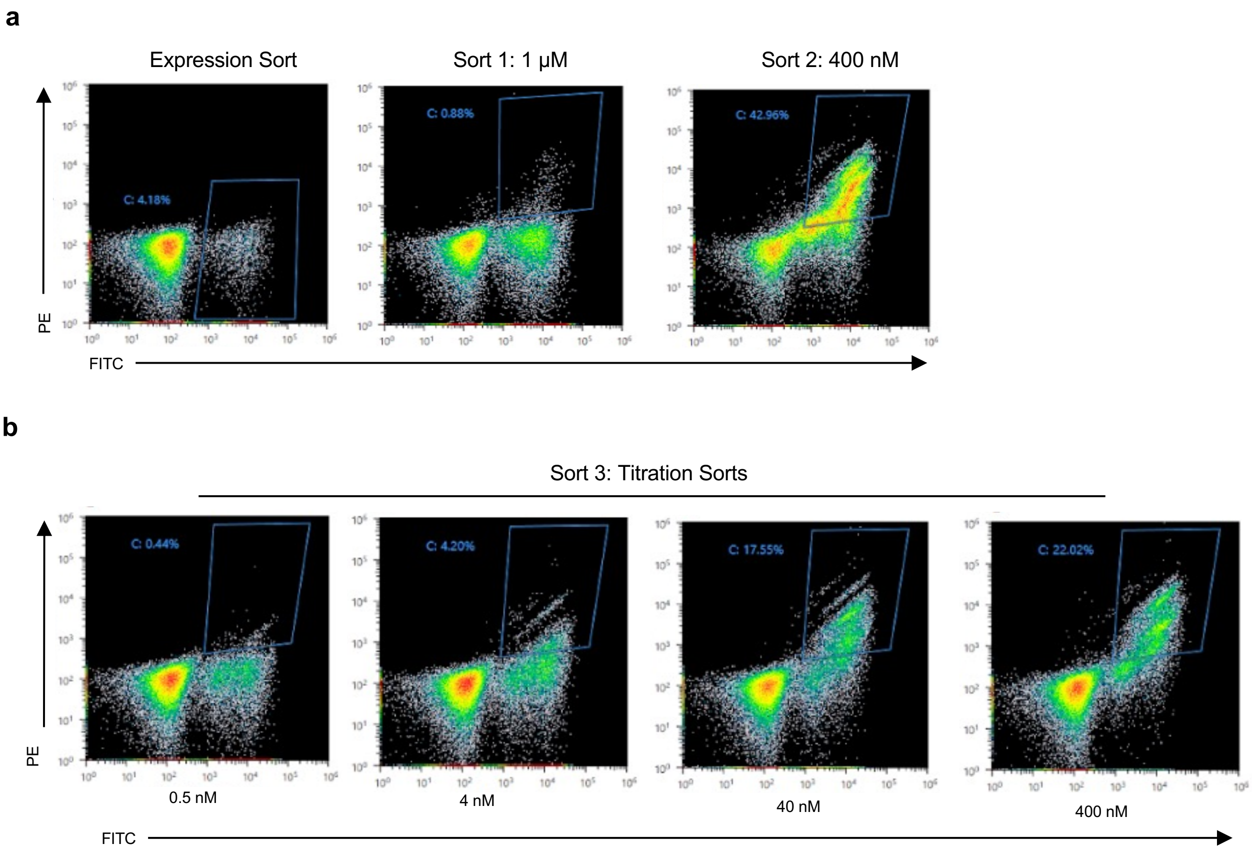

Figure S1: Yeast surface display

a) Initial sequential flow cytometry enrichment of yeast expressing DNMT3A binders, followed by addition of 1 μM and 400 nM of FLAG-tagged DNMT3A after subsequent enrichments.

b) Final round of flow cytometry titration sorts showing binder enrichment at 0.5 nM, 4 nM, 40 nM, and 400 nM of FLAG-tagged DNMT3A.

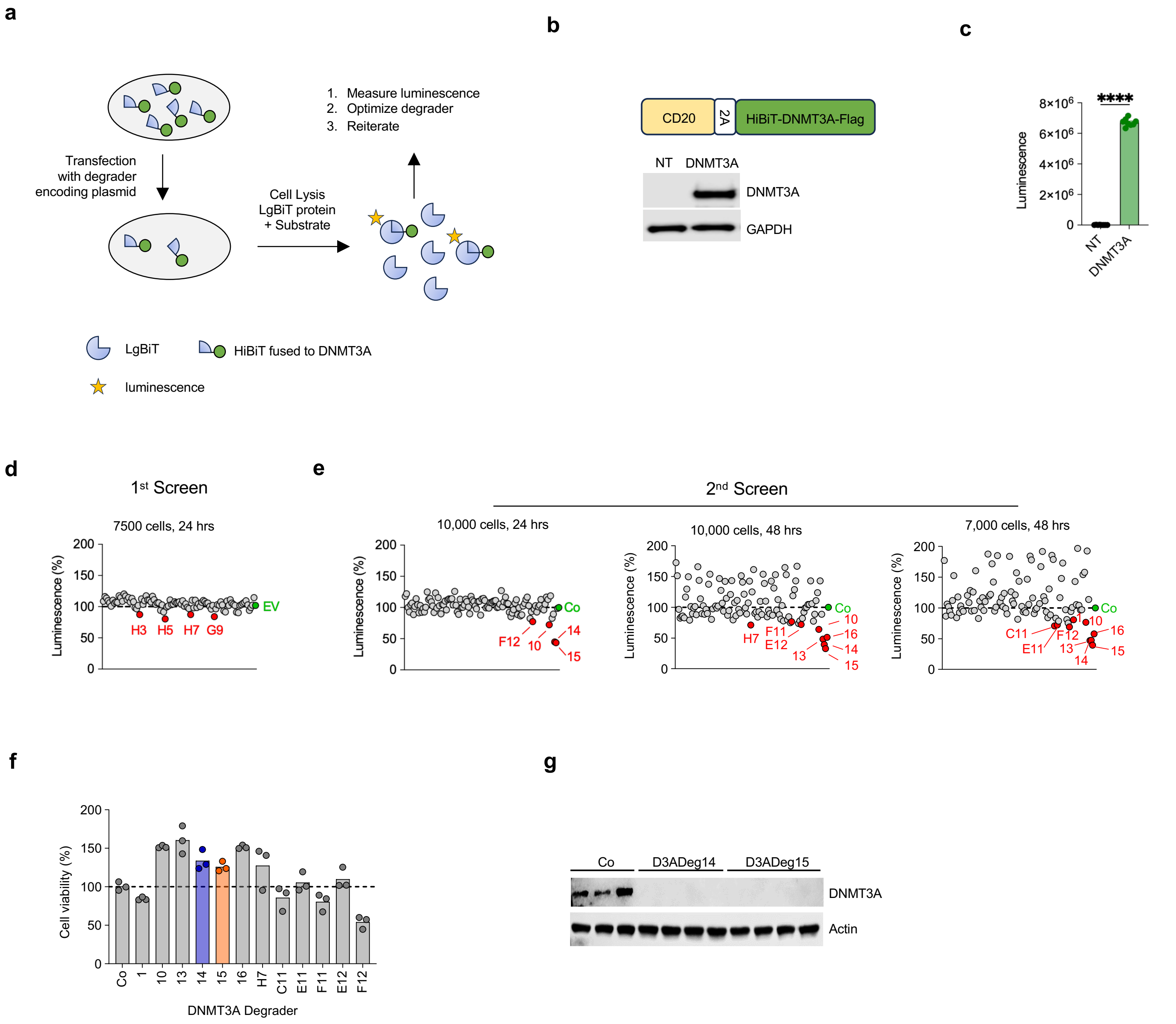

Figure S2: DNMT3A bioPROTAC screen

a) Screen schematic.

b) Retroviral construct encoding CD20 and HiBiT DNMT3A fusion. Western blot of non-transduced (NT) and transduced 293T cells (D3A 293T cells).

c) Bioluminescence assay comparing NT and transduced 293T cells.

d) Result of first screen; Co: control (empty vector).

e) Result of second screen. Screen was conducted at two cell concentrations.

f) Cell viability of potential hits shown in Figure 1c.

g) Detection of DNMT3A by Western blot in D3A 293T cells transfected with D3A.Deg14 and D3A.Deg15; Co: control (empty vector).

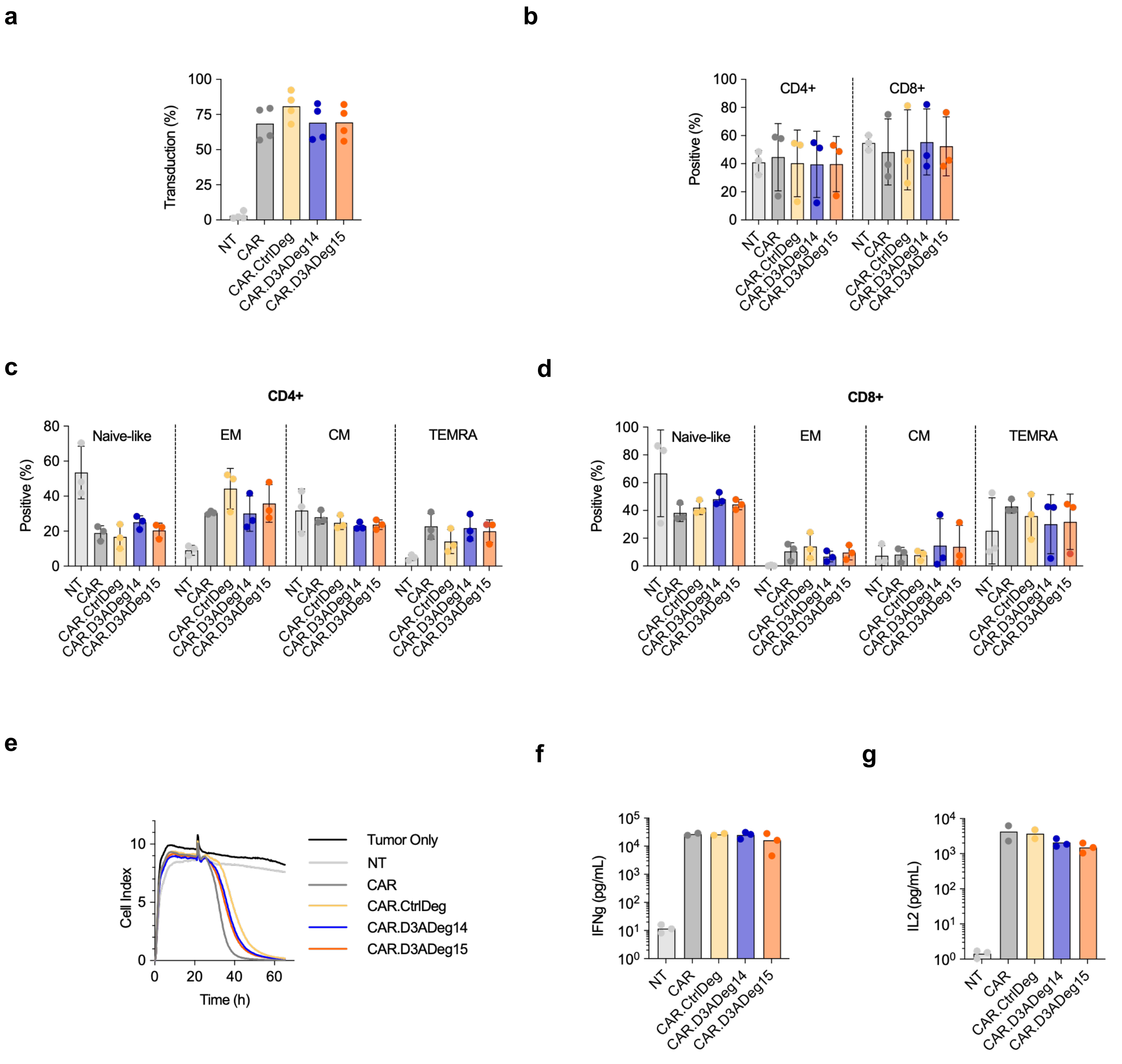

Figure S3: Characterization of DNMT3A bioPROTAC expressing CAR T-cells

a) Transduction efficiency at day 7 post-transduction assessed by flow cytometry for CAR (n=4)

b-d) Immunophenotype of CAR T cells assessed by flow cytometry; frequency of **(b)** $CD4^+$ and $CD8^+$ T cells. T cell differentiation state of **(c)** $CD4^+$ **(d)** and $CD8^+$ **(d)** T cells. Effector memory (EM): $CCR7^-$ $CD45RA^-$, central memory (CM: $CCR7^+$ $CD45RA^-$), naïve-like: $CCR7^+$ $CD45RA^+$, and T-effector memory re-expressing CD45RA (TEMRA): $CCR7^-$ $CD45RA^+$, (n=3).

e) Cytolytic activity of NT and indicated CAT T cell populations measured using xCELLigence real-time cell analysis (RTCA). B7-$H3^+$ LM7 tumor cells were co-cultured at an effector-to-target ratio of 2:1. One representative donor is shown, performed in triplicate.

f-g) NT and indicated CAR T cell populations were co-cultured with LM7 cells at a 2:1 E:T ratio. After 24 hours, **f)** IFNγ and **g)** IL-2 concentrations in the media were quantified by ELISA (n=2-3).

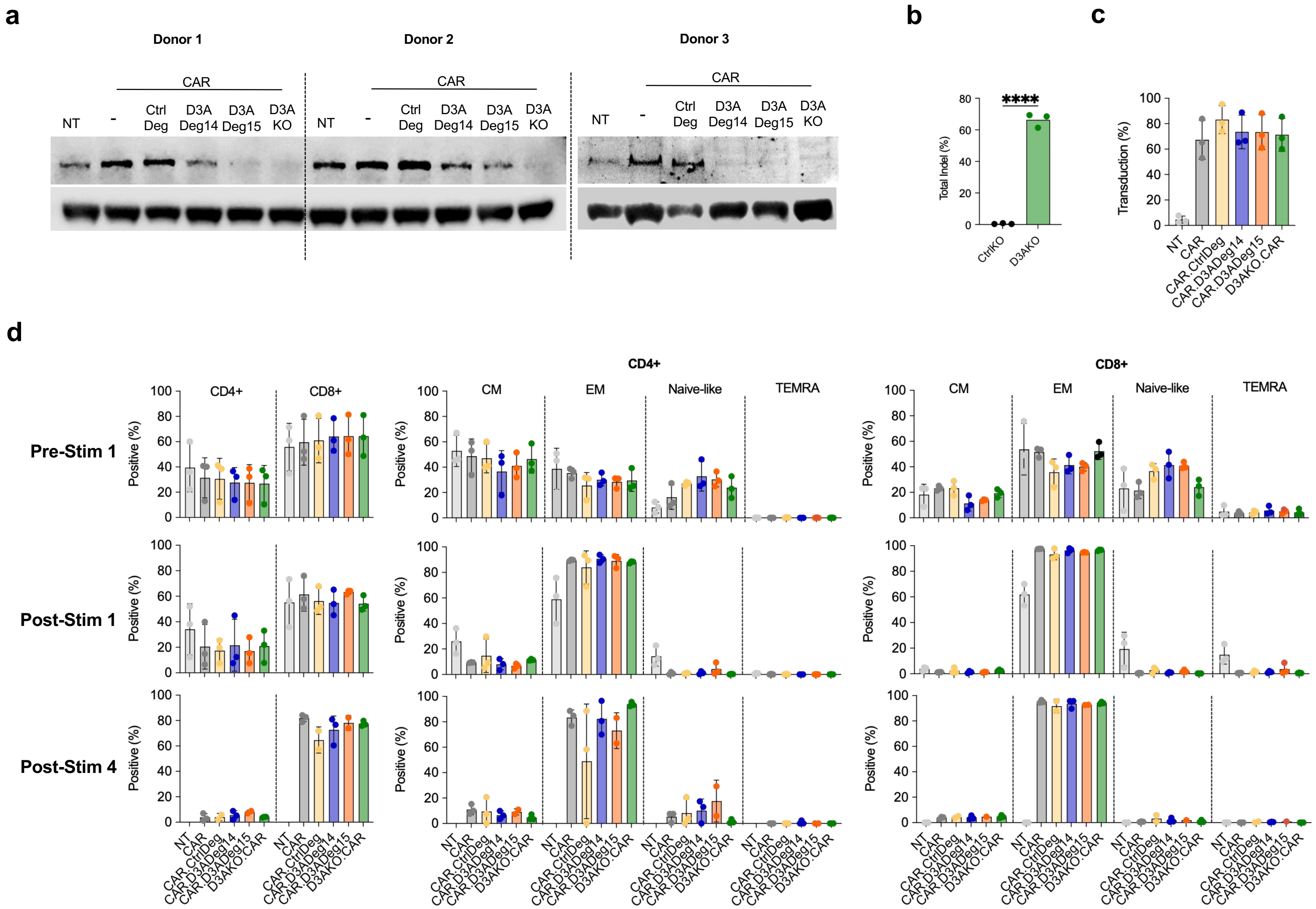

Figure S4: Characterization of CAR T-cell populations

a) Western blot confirming DNMT3A degradation (CAR.D3ADeg14, CAR.D3ADeg15) and deletion (D3AKO.CAR) on day 7 post-transduction (n=3).

b) INDEL analysis of DNMT3A KO T cells (****P < 0.0001, n=3).

c) Transduction efficiency of NT and indicated CAR T-cell populations control assessed on day 7 post-transduction by flow cytometry.

d) Immunophenotype of CAR T cells assessed by flow cytometry pre-stimulation (stim) 1, and after (post-stim) 1 and 4 stimulations (n=3).

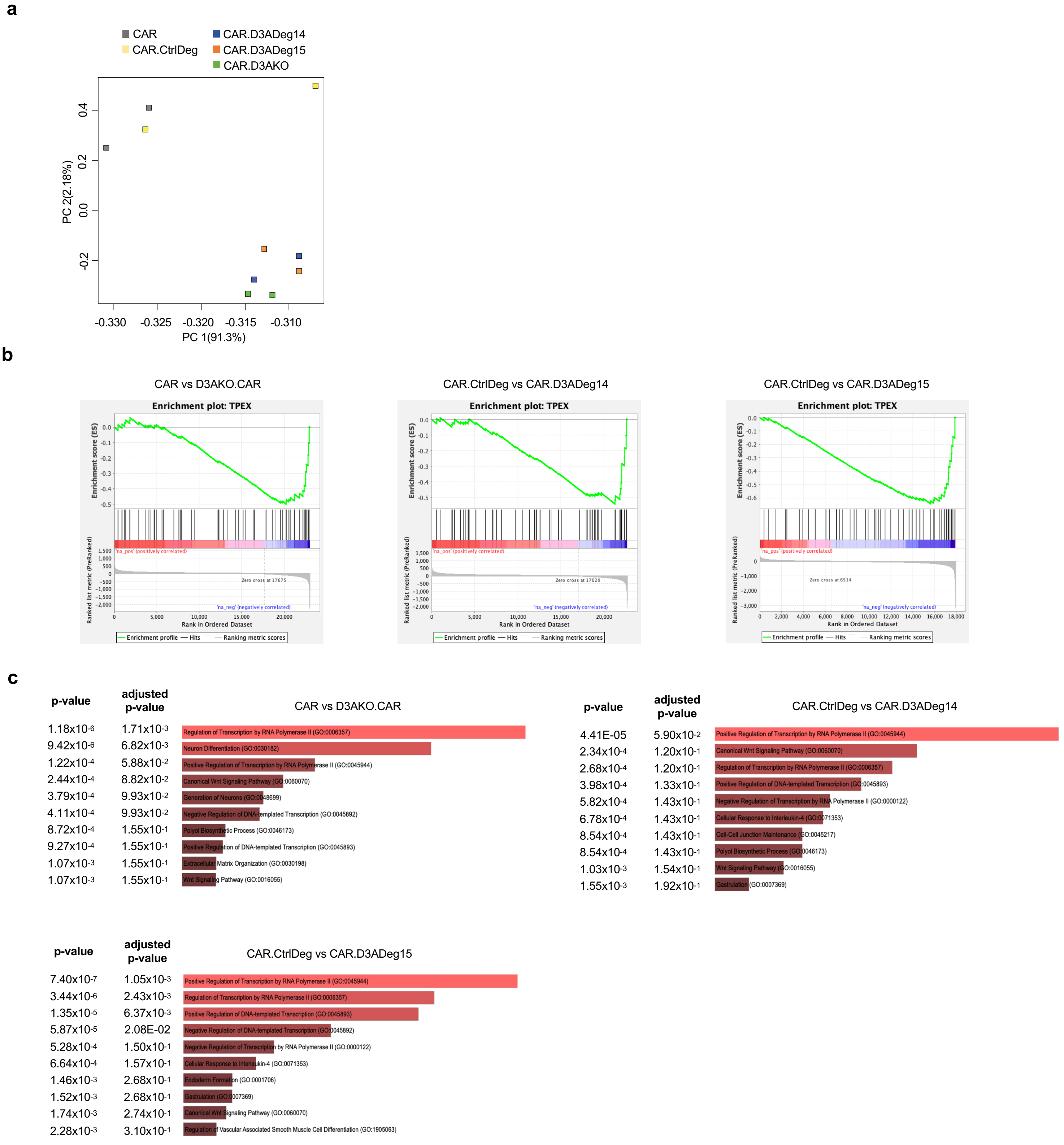

Figure S5: DNMT3A bioPROTACs preserve CAR T-cell plasticity

Figure S4 shows supplementary data analysis for the data shown in Figure 2a-h.

a) Principal Component Analysis (PCA) of DMR-based methylation profiles showing variance across CAR, CAR.CtrlDeg, CAR.D3ADeg14, CAR.D3ADeg15, and D3AKO.CAR conditions.

b) GSEA of DMRs after 4th repeat stimulation using a gene signature associated with progenitor exhausted T cells (TPEX). The following comparison are shown: CAR vs D3AKO.CAR, CAR.CtrlDeg vs CAR.D3ADeg14, and CAR.CtrlDeg vs CAR.D3ADeg15.

c) GO analysis of DNMT3A-regulated genes after 4th repeat stimulation. The following comparison are shown: CAR vs D3AKO.CAR, CAR.CtrlDeg vs CAR.D3ADeg14, and CAR.CtrlDeg vs CAR.D3ADeg15 with p-values, Fisher's exact test.
